# Infantile Paralysis

**Published:** 1898-04-02

**Authors:** 


### INFANTILE PARALYSIS.

In a clinical lecture recently delivered at the Queen's Square Hospital, Dr. Buzzard related several cases illustrating the infective origin of infantile paralysis. The matter is one of very great interest; for, as Dr. Buzzard says, infantile paralysis is a peculiarly cruel disease. It rarely has the mercy to kill the patient, but usually condemns its victims to a prolonged existence embittered by more or less helplessness and deformity. Moreover, our present means of dealing with this disease, when once produced, are so trifling as scarcely to deserve serious mention.

The preliminary fever which precedes the onset of the paralysis is well known; but the suggestion is made that this is not so much a first stage as an independent disease, and that it is by dint of some destructive toxin, elaborated during this fever, or by its specific (microbic?) cause, that the paralysis is produced, much as post-diphtheritic paralysis is caused by the toxins elaborated in diphtheria. Several small "epidemics" of infantile paralysis are referred to, and it is pointed out that alongside of those cases in which febrile attacks ended in typical paralysis, other cases occurred in which febrile attacks of apparently the same character passed away without producing this disastrous result. It is very desirable then, that, whenever a case of infantile paralysis is met with, the character of any febrile ailments occurring in the same locality and about the same time should be carefully studied; for if it should be possible to discover and differentiate a specific infectious fever as the necessary antecedent of infantile paralysis, one might indulge the hope that science might be able to introduce some effective method of combating the toxic agent before it has produced those crippling results which, when they have once been set up, are so little amenable to treatment.

